# First report of naturally infected *Aedes aegypti* with chikungunya virus genotype ECSA in the Americas

**DOI:** 10.1371/journal.pntd.0005630

**Published:** 2017-06-14

**Authors:** André Luis Costa-da-Silva, Rafaella Sayuri Ioshino, Vivian Petersen, Antonio Fernando Lima, Marielton dos Passos Cunha, Michael R. Wiley, Jason T. Ladner, Karla Prieto, Gustavo Palacios, Danuza Duarte Costa, Lincoln Suesdek, Paolo Marinho de Andrade Zanotto, Margareth Lara Capurro

**Affiliations:** 1 Departamento de Parasitologia, Instituto de Ciências Biomédicas, Universidade de São Paulo, São Paulo, SP, Brasil; 2 Instituto Nacional de Ciência e Tecnologia em Entomologia Molecular, INCT-EM, Rio de Janeiro, Rio de Janeiro, Brazil; 3 Laboratório de Parasitologia, Instituto Butantan, São Paulo, Brazil; 4 Fundação de Saúde Parreiras Horta/LACEN, Aracaju, Sergipe, Brazil; 5 Departamento de Microbiologia, Instituto de Ciências Biomédicas, Universidade de São Paulo, SP, Brasil; 6 Center for Genome Sciences, United States Army Medical Research Institute of Infectious Diseases, Frederick, Maryland, United States of America; 7 Programa de Pós-graduação do Instituto de Medicina Tropical, Universidade de São Paulo, SP, Brasil; Florida Department of Health, UNITED STATES

## Abstract

**Background:**

The worldwide expansion of new emergent arboviruses such as Chikungunya and Zika reinforces the importance in understanding the role of mosquito species in spreading these pathogens in affected regions. This knowledge is essential for developing effective programs based on species specificity to avoid the establishment of endemic transmission cycles sustained by the identified local vectors. Although the first autochthonous transmission of Chikungunya virus was described in 2014 in the north of Brazil, the main outbreaks were reported in 2015 and 2016 in the northeast of Brazil.

**Methodology/Principal findings:**

During 5 days of February 2016, we collected mosquitoes in homes of 6 neighborhoods of Aracaju city, the capital of Sergipe state. Four mosquito species were identified but *Culex quinquefasciatus* and *Aedes aegypti* were the most abundant. Field-caught mosquitoes were tested for Chikungunya (CHIKV), Zika (ZIKV) and Dengue viruses (DENV) by qRT-PCR and one CHIKV-infected *Ae*. *aegypti* female was detected. The complete sequence of CHIKV genome was obtained from this sample and phylogenetic analysis revealed that this isolate belongs to the East-Central-South-African (ECSA) genotype.

**Conclusions:**

Our study describes the first identification of a naturally CHIKV-infected *Ae*. *aegypti* in Brazil and the first report of a CHIKV from ECSA genotype identified in this species in the Americas. These findings support the notion of *Ae*. *aegypti* being a vector involved in CHIKV outbreaks in northeast of Brazil.

## Author summary

The chikungunya outbreaks have become one of the main public health problems in the Northeast Region of Brazil. Since the chikungunya virus (CHIKV) was recently introduced in this country, finding vector associations involved in its transmission to the vertebrate host is primordial to better design efficient intervention against mosquitoes. In the present study, field-caught mosquitoes in Aracaju city, Sergipe State, Brazil were evaluated for the presence of CHIKV and other two endemic arboviruses (Zika and Dengue viruses) in this region. We described here the first identification of the East-Central-South-African (ECSA) CHIKV genotype naturally infecting *Aedes aegypti* mosquitoes in Brazil. This work suggests the involvement of this species in CHIKV outbreaks in Northeast Region of this country and reveals a possible interaction between this vector and the recently introduced CHIKV ECSA genotype.

## Introduction

Chikungunya viral disease is caused by an arbovirus (*Alphavirus* genus) from *Togaviridae* family [1,2]**.** The most frequent symptoms during human infection are fever and joint pain, but headache, muscle pain, joint swelling and rash are also observed [3,4]. Chikungunya virus (CHIKV) is transmitted to humans by *Aedes* mosquitoes in sylvatic (animal-mosquito-man) [5] or urban transmission cycles (man-mosquito-man) [6]. This virus is endemic in Africa and Asia, and has spread to several European countries and more recently to America [7]. In Brazil, the first autochthonous case was described in 2014 in Amapá, a state in the North Region [8]. Since then, CHIKV has expanded its distribution in Brazil and 13,236 cases were confirmed in 2015, most of them occurring in states within the Northeast Region [9–11]**.**

Three CHIKV genotypes have been identified since its discovery in 1952 [2]. The East-Central-South-African (ECSA) and West African genotypes circulate in sub-Saharan Africa, whereas *Ae*. *aegypti* is the vector of the Asian genotype in human urban transmission cycles in Southeast Asia and in the Americas. Asian and ECSA genotypes were described in Brazil concomitantly with autochthonous cases caused by both genotypes [12]. Currently, all Brazilian states are infested by both of the main CHIKV vectors, *Ae*. *aegypti* and *Ae*. *albopictus* [13,14]. The presence of both species raises concern about the establishment of sustained CHIKV outbreaks throughout the entire country [15]. Although neither of these vectors have yet been found to be naturally infected with CHIKV in Brazil, it is known that Brazilian populations of both species are highly competent vectors for CHIKV when infected under laboratory conditions [16]. Moreover, it is not known if other *Culicidae* species occurring in urban area in Brazil are capable to transmit CHIKV. Therefore, entomological surveillance is essential for understanding the role of vector species in CHIKV transmission, especially in new endemic countries such as Brazil. Recently, Aracaju, a Northeast Region city and the capital of Sergipe state with 571,149 inhabitants [17], has experienced an increase in CHIKV cases since the detection of the first autochthonous transmission in 2015. According to the State Department of Health from the Sergipe government, the highest number of CHIKV cases in Aracaju has been reported in 2016, being 6,810 suspected and 4,743 confirmed until epidemiological week 33. The same epidemiological bulletin reported 30 confirmed cases for Zika virus (ZIKV) and 1,457 for Dengue virus (DENV) in 2016 [18].

In this alarming scenario, we hypothesize that the city of Aracaju is a potential site for detecting vector species naturally infected by CHIKV, ZIKV and DENV. During the third week of February 2016, we collected adult mosquitoes, inside and outside homes situated in urban areas of Aracaju, where residents were complaining of symptoms consistent with CHIKV or related arboviruses (DENV and ZIKV). Our results revealed that the ECSA genotype of CHIKV is naturally infecting *Ae*. *aegypti* in the Americas and showed the first identification of a field-caught mosquito infected with CHIKV in Brazil. These findings confirm the vectorial capacity of *Ae*. *aegypti* for transmitting CHIKV and suggests that this species is an important CHIKV vector which can be involved in the recent outbreaks since this virus was introduced in this country.

## Material and methods

### Fieldwork and identification

During a multidisciplinary coordinated investigation involving the ZIKV São Paulo task force (Zika Network) and the Central Public Health Laboratories (LACEN—Aracaju, Sergipe, Brazil), medical interviews, clinical diagnosis and mosquito collections were performed during one week in Aracaju. Houses in six different neighborhoods were selected based on the locations of clinically ill patients (suspected symptoms of arboviral infection) provided by LACEN. All neighborhoods were mostly residential, composed by heterogeneous house sizes of very low- to low-income, except by São José neighborhood, with middle-income dwellings. After an authorized employee from LACEN obtained permission of the residents, mosquitoes were collected by the entomological team. Peridomiciliary and intradomiciliary areas (all the rooms) of these houses and adjacent residences were aspirated during the morning after the dawn or afternoon until the dusk period using vacuum aspirators manufactured by Horst Armadilhas. Captured mosquitoes were placed into a collection vial previously identified by date and neighborhood and for each home inspected, one vial was used.

The collected mosquitoes were ice anesthetized and sorted by species using a morphological identification key as reference when necessary [19], sex and collected region. Males were pooled in 4 to 11 individuals of the same species and both engorged and non-engorged females were individually divided into head, thorax and abdomen using a McPherson-Vannas Scissors #501234 (World Precision Instruments, Sarasota, FL) to avoid contamination from possible blood inside the mosquito midgut. Samples were stored individually into a 1.5 mL microtube and immediately frozen at -80°C for further analyses.

### RNA extraction of samples

Total RNA was extracted from each individual female thorax and from pooled whole male mosquitoes using QIAmp Viral RNA Mini Kit (Qiagen, USA) following manufacturer’s recommendations. About 5 to 10 μL of extracted thoracic RNA from each female was pooled in a species-specific manner and the remaining volume of each RNA sample was stored at -80°C for subsequent individual confirmations and further analyses.

### One step qRT-PCR detection of arbovirus

The species-specific RNA pools were tested for CHIKV, ZIKV and DENV1-4 presence using QuantiTect Probe RT-PCR kit (Qiagen, USA) and Mastercycler Realplex 2 thermocycler (Eppendorf, Germany). Primers/probes used for detection of emerging arbovirus were previously described for DENV1-4 serotypes (DEN FP and DEN RP primers and DEN P probe) [20], ZIKV (ZIKV 835 and ZIKV 911c primers and ZIKV 860 probe) [21] and CHIKV (CHIKV FP1 and CHIKV RP1 primers and CHIKV P2 probe) [22]. CHIKV primers and probe applied in this study detect the 3 CHIKV genotypes.

The one step qRT-PCR conditions, including total RNA sample volume and cycling for ZIKV and CHIKV detection reactions were previously described [2]. For DENV detection, 20μL reactions were used as following: 10 μL of QuantiTect Probe RT-PCR Master mix, 0.25 μL of QuantiTect RT mix, 1 μL of 10 μM of each primer, 0.4 μL of 10 μM probe, 2.4 μL of DNA RNA free water and 5 μL of total RNA sample. The thermocycler conditions for DENV were 50°C for 20 min and 95°C for 15 min followed by 45 cycles of 95°C for 15 sec, 60°C for 15 sec and 40°C for 30 sec. For each qRT-PCR experiment, a positive control consisting of a specific viral RNA (DENV2, ZIKV or CHIKV) was added for cDNA synthesis and detection validation. Each sample, as well as negative (DEPC treated water as template) and positive controls, were analyzed in technical duplicates.

For positive pools, individual RNA samples were retested to confirm which mosquitoes from the positive pool were infected.

### Sequencing of CHIKV

*Ae*. *aegypti* RNA from CHIKV positive female was prepared for NGS sequencing using a sequence-independent single-primer amplification (SISPA) method as described previously [23]. Libraries were sequenced with an Illumina MiSeq desktop sequencer using a version 3 kit (2 x 150 cycles). Additionally for mosquito sample that was positive for CHIKV we used the Illumina TruSeq RNA Access enrichment method to get coding-complete genomes as described previously [24]. CHIKV specific probes were designed against Brazilian isolate KP164569.

### Phylogenetic analysis

For phylogenetic characterization of sequenced CHIKV detected in positive mosquitoes from Sergipe-Brazil, additional CHIKV sequences from Brazil and other countries, representing all known genotypes, were recovered from Genbank (http://www.ncbi.nlm.nih.gov) (S1 Table). All sequences used in this work are presented in the format: genotype/accession number/country/year of isolation in the phylogenetic tree. Sequences were aligned using Clustal X2 [25] and the alignment was manually edited using jalview.

Viral phylogenies based on full-length nucleotide sequences were estimated using Maximum Likelihood (ML) implemented in FastTree 2 [26] and Bayesian Inference (BI) analysis with BEAST package v.1.8.2 [27], using the general time-reversible with gamma-distributed rate variation substitution model (GTR+G), as described by Akaike’s information criterion (AICc) in jModelTest 0.1 [28]. For the BI, a relaxed lognormal molecular clock model and the nonparametric Skygrid coalescent model were employed. The evolutionary analysis were computed using two independent runs for 100 million MCMC steps and the convergence of the MCMC chains was inspected using TRACER v.1.6 (http://tree.bio.ed.ac.uk). Posterior trees were summarized discarding the first 10% of the sampled trees and choosing the Maximum Clade Credibility (MCC) was summarized using TreeAnnotator v.1.8.2. The final trees were then visualized and plotted using FigTree v.1.4.2 (http://tree.bio.ed.ac.uk).

## Results

### Collected species and frequency of occurrence

**Fig 1:**
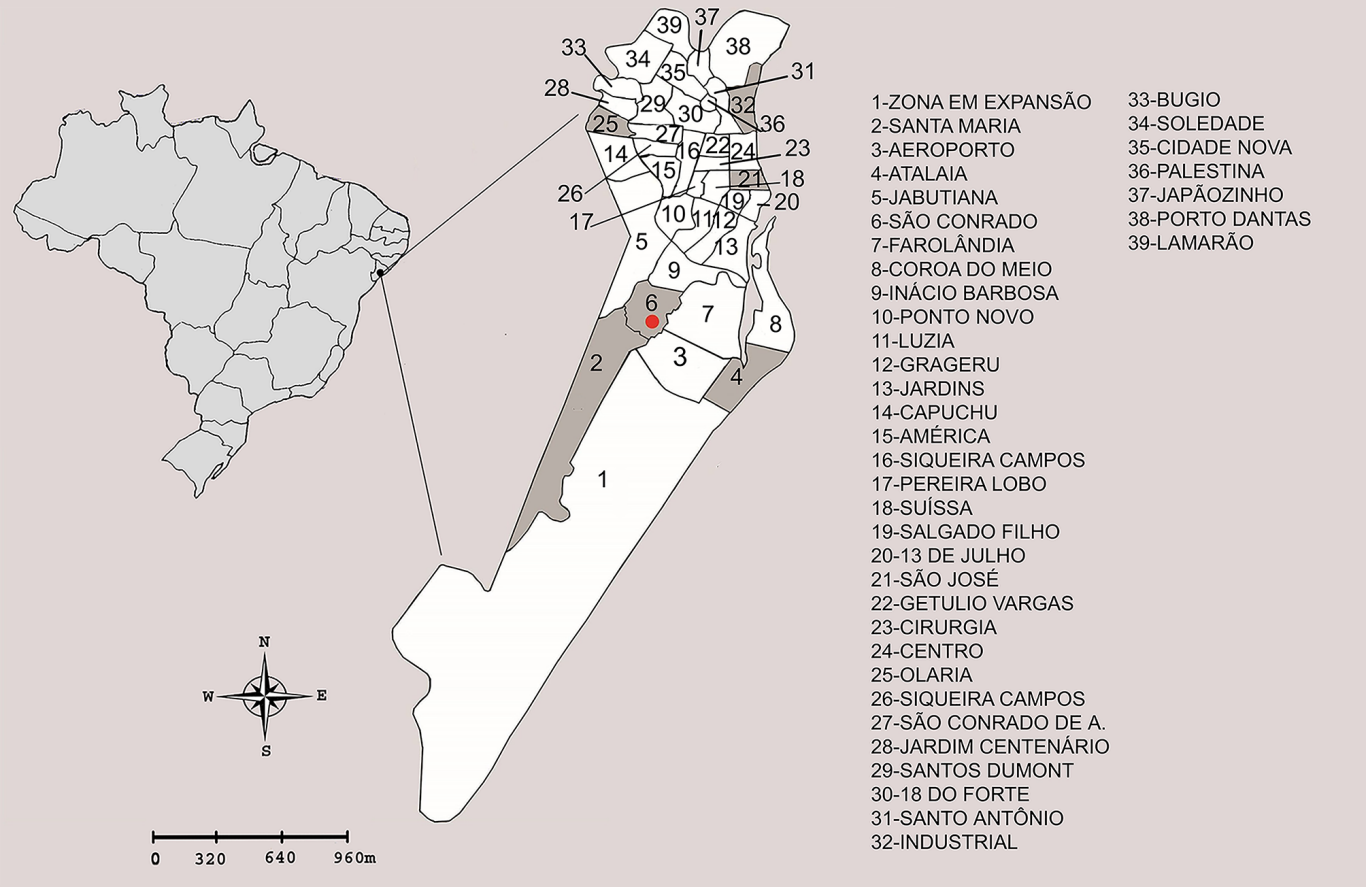
Map of the Aracaju city and neighborhood boundaries. The neighborhoods where mosquitoes were collected are shaded in gray. The red dotted neighborhood is where CHIKV-positive *Ae*. *aegypti* were collected.

Six neighborhoods were inspected in Aracaju city during February 16^th^ to 20^th^, 2016 (Fig 1).

A total of 248 mosquitoes were collected in 39 properties and we identified 4 species during the triage procedure and identification (Table 1).

**Table 1:** Neighborhoods, inspected properties and mosquito species identified in Aracaju city, Sergipe state.

Neighborhood visited	Inspected properties	Number of species	Number of individuals collected by species			
			*Aedes aegypti*	*Culex quinquefasciatus*	*Aedes scapularis*	*Aedes taeniorhynchus*
Atalaia	5	3	11	56	1	0
São José	4	2	4	27	0	0
Industrial	14	3	13	44	0	1
Olaria	8	2	7	18	0	0
Santa Maria	1	1	0	31	0	0
São Conrado	7	4	15	18	1	1
**Total**	39	4	50	194	2	2

*Culex quinquefasciatus* were the most frequently collected species (78.2%,194/248), 3.88 times more represented than *Ae*. *aegypti* (20.2%, 50/248). Two other species, *Ae*. *scapularis* and *Ae*. *taeniorhynchus* were also captured, but with the same low proportion (0.8%, 2/248 each) in relation to the total number of mosquitoes collected.

Fifteen properties presented the co-occurrence of multiple species. The co-occurrence of *Ae*. *aegypti*-*Cx*. *quinquefasciatus* was the most registered, but we also found other combinations of concurrences as well. For instance, *Cx*. *quinquefasciatus*/*Ae*. *aegypti*/*Ae*. *taeniorhynchus* or *Ae*. *scapularis* were found in the same home. However, the co-occurrence of more than three species in the same property was not observed.

### Detection of CHIKV, ZIKV and DENV1-4

We tested the mosquitoes for DENV, ZIKV and CHIKV because Aracaju is endemic for DENV and human cases of ZIKV and CHIKV infection were recently confirmed in this city in 2016. All eight male and fifteen female pools of *Cx*. *quinquefasciatus*, as well as the one *Ae*. *scapularis* female pool and the *Ae*. *taeniorhynchus* female pool, were negative for CHIKV, ZIKV and DENV serotypes 1–4. Together, these pools represented a total of 198 mosquitoes (Table 2). Negative results were also observed for ZIKV and DENV1-4 detection in all male and female *Ae*. *aegypti* pools. However, one *Ae*. *aegypti* female pool was positive for CHIKV from a total of thirteen pools tested. All male pools presented negative for CHIKV (Table 2).

**Table 2:** Collected species and results of one step qRT-PCR diagnostics for Dengue, Chikungunya and Zika viruses.

Mosquito species analyzed	FEMALES					
	Number of mosquitoes	Pools tested	Positive pools			CHIKV positive mosquitoes
			DENV1-4	CHIKV	ZIKV	
*Aedes aegypti*	38	10	0	1	0	1
*Culex quinquefasciatus*	111	15	0	0	0	0
*Aedes scapularis*	2	1	0	0	0	0
*Aedes taeniorhynchus*	2	1	0	0	0	0
Mosquito species analyzed	MALES					
	Number of mosquitoes	Pools tested	Positive pools			
			DENV1-4	CHIKV	ZIKV	
*Aedes aegypti*	12	3	0	0	0	
*Culex quinquefasciatus*	83	8	0	0	0	
*Aedes scapularis*	0	0	0	0	0	
*Aedes taeniorhynchus*	0	0	0	0	0	

Following the pool analysis, we tested the individual thoracic samples that composed the CHIKV positive pool. We were able to detect one positive female, confirming our initial analysis (Table 2). The positive female was collected in São Conrado neighborhood (Fig 1), and the mean Ct values obtained in qRT-PCR was 21.76 for the individual thorax from the positive sample. Therefore, of thirty-eight females and twelve males of *Ae*. *aegypti* analyzed, only one female was infected with CHIKV.

### Sequencing and phylogeny of CHIKV

The total RNA from CHIKV positive sample was selected for sequencing using Illumina sequencing platform. The whole sequence of the CHIKV genome was obtained and deposited in GenBank with accession number: KY055011.

Identity analysis of the whole CHIKV genome generated in this study with representative sequences of all known CHIKV genotypes obtained in GenBank (S1 Table) had percentages identities ranging from 84.24% to 99.99%, while between CHIKV sequence previously isolated in 2014–2015 in Bahia state showed percentages identities ranging from 99.91% to 99.99% (S2 Table). The mutations previously associated with CHIKV fitness increase in *Ae*. *aegypti* and *Ae*. *albopictus* [29,30] were not detected in envelope protein 1 (E1) from sequenced CHIKV.

**Fig 2:**
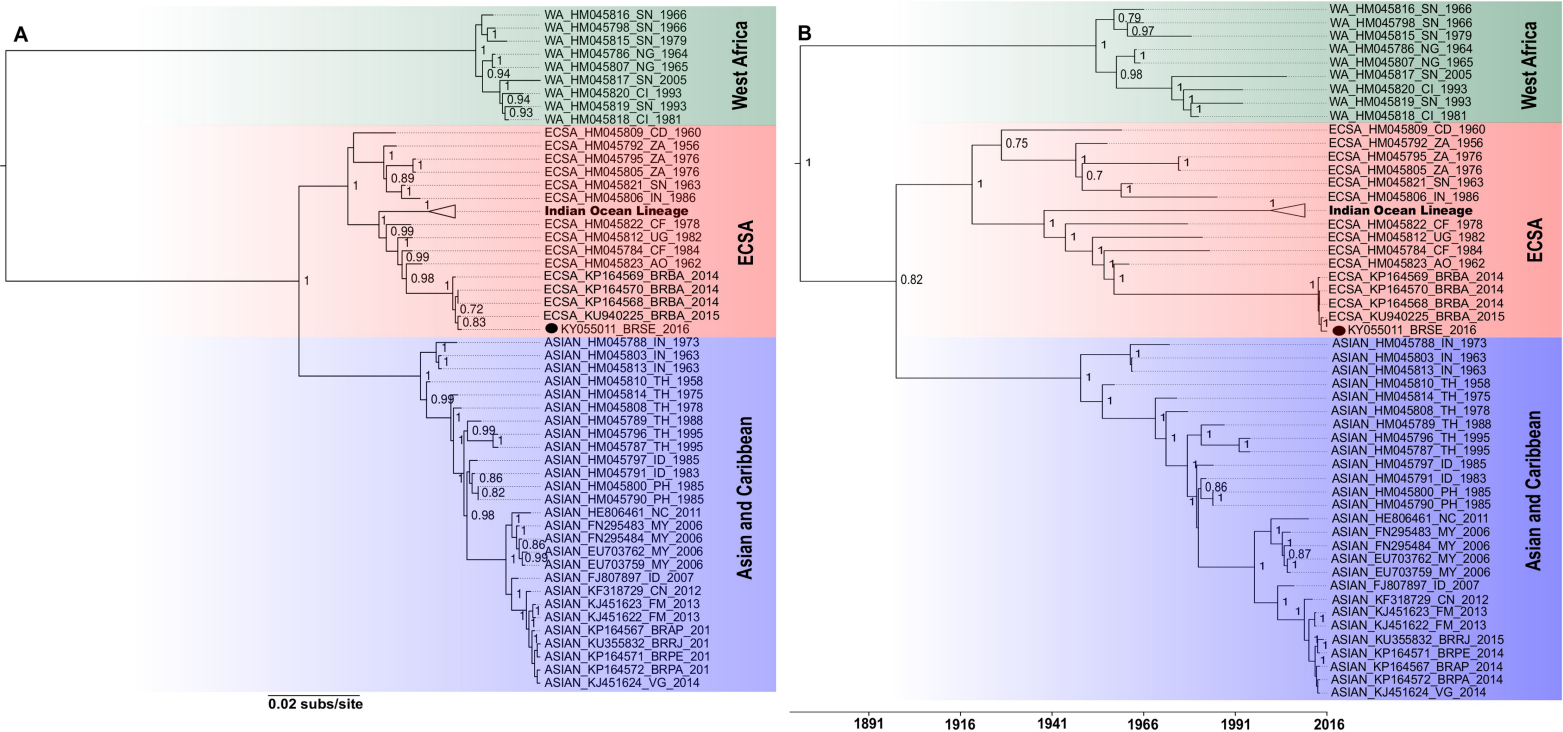
Evolutionary relationship between the three genotypes of CHIKV. A. Maximum likelihood phylogenetic tree of the CHIKV genomic sequence (KY055011_BRSE_2016) obtained from positive field-caught *Ae*. *aegypti*. The numbers on the branches represent the bootstrap values. B. Temporal maximum clade credibility tree of the CHIKV genomic sequence (KY055011_BRSE_2016) obtained from positive field-caught *Ae*. *aegypti*. The numbers on the branches represent the posterior probability values.

Maximum likelihood analysis and Bayesian Inference comparing the CHIKV sequence isolated in Sergipe with sequences previously characterized (S1 Table) produced trees with similar topologies (Fig 2A and 2B, respectively).

The CHIKV-mosquito sequence was closely-related to other sequences from Bahia-Brazil isolated in 2014–2015 and with sequences from Africa, characterized as ECSA genotype (Fig 2). The consistency of the results is supported by the high *bootstrap* values and posterior probability observed in the trees (Fig 2A and 2B, respectively).

## Discussion

The identification of the vectors in endemic areas is essential to better design surveillance programs and defines efficient vector control strategies. In Brazil, it is well known that *Ae*. *aegypti* is the main DENV vector and although *Ae*. *albopictus* is also a potential vector in this country, ecological characteristics and adaptations favor the first species to sustain DENV outbreaks in urban areas [31]. Nevertheless, the recent introductions of CHIKV and ZIKV in Brazil and severe epidemics reported, have raised fundamental questions regarding the establishment of viral circulation in relation to the mosquito species occurring in endemic places [32].

A recent study showed that American populations of *Ae*. *aegypti* and *Ae*. *albopictus*, including Brazilian ones, are highly competent to transmit different CHIKV genotypes when orally infected in the laboratory [16]. Based on their results, the authors highlighted the imminent risk for an expansion of CHIKV outbreaks in tropical and subtropical areas of this continent [16]. In fact, *Ae*. *aegypti* transmitting CHIKV Asian genotype in the Americas was first described in Mexico, during an outbreak in Ciudad Hidalgo, Chiapas State [33].

CHIKV was first isolated in Brazil from infected patients in 2014 and the circulation of two genotypes was observed: Asian genotype in Oiapoque (Amapá state), North Region of Brazil and ECSA genotype in Feira de Santana (Bahia state), Northeast Region of Brazil [12]. It is important to highlight that Aracaju is in the Northeast Region and is approximately 300 km away from Feira de Santana. Coherently, we report here the first detection of a natural infection of *Ae*. *aegypti* mosquito by CHIKV genotype ECSA in Brazil and the Americas.

In the present study, *Ae*. *albopictus* was not found in the inspected houses although this species is endemic in Aracaju city [14]. However, we found two unusual species intra domiciliary, *Ae*. *scapularis* and *Ae*. *taeniorhynchus*, but the occurrence was much less frequent than *Ae*. *aegypti* and *Cx*. *quinquefasciatus*. The collected females from *Ae*. *scapularis* (n = 2) and *Ae*. *taeniorhynchus* (n = 2) were negative for ZIKV, CHIKV or DENV 1–4 serotypes. These females were engorged suggesting they are biting humans inside their homes. Moreover, it is known that *Ae*. *scapularis* presents vectorial competence for Rocio encephalitis, Ilheus and Melao viruses [34–36] while *Ae*. *taeniorhynchus* for Venezuelan equine encephalitis and West Nile viruses [37,38]. Nevertheless, it is unknown if these mosquito species are competent vectors in Brazil for the three arboviruses assayed in this study and if they can act as secondary vectors during outbreaks.

Recently, *Ae*. *aegypti* was shown to be a ZIKV vector in Rio de Janeiro city. In this study ZIKV-positive *Cx*. *quinquefasciatus* or *Ae*. *albopictus* were not identified [39]. We found *Ae*. *aegypti* as a potential vector of CHIKV in Aracaju city, as seen in outbreaks already reported elsewhere [5,40–42]. None of the other species examined were positive for CHIKV, ZIKV or DENV serotypes 1–4, mainly *Cx*. *quinquefasciatus* that was abundant in our sampling effort.

The surveillance of the *Aedes* mosquitoes should be expanded in order to prevent new CHIKV outbreaks in Brazil, since this country presents adequate conditions for the establishment of an endemic situation, which can also exposes other countries at risk. The mutations that enhance the fitness of the CHIKV genotype ECSA in *Aedes* mosquitoes were previously described [29,30] and they were not found in the genotype characterized in our study. However, new mutations that improve vector competence can be acquired since the ECSA genotype is being detected in other regions in Brazil [43], which suggests that different populations of *Aedes* mosquitoes are interacting with this recently introduced genotype, mainly *Aedes aegypti* in urban areas.

Our findings constitute the first description of *Ae*. *aegypti*-CHIKV genotype ECSA interaction in Brazil. These results reinforce the role of this species as an important vector of CHIKV in urban areas of northeast regions in Brazil.

## Supporting information

S1 Table Available CHIKV sequences information. The GenBank accession number of the sequences used in phylogenetic analyzes.
(XLSX)

S2 Table Sequences identity between Brazilian CHIKV and representative sequences of all known genotypes based on the coding regions. The values in bold correspond to the identity value and the values in italic correspond to the number of polymorphic sites between the sequences.
(XLSX)
